# Early Outcomes of Extended Hepatectomy: An Experience from the University Hospital of Nepal

**DOI:** 10.7759/cureus.34036

**Published:** 2023-01-21

**Authors:** Deepak Sharma, Krishna M Adhikari, Narendra Maharjan, Sumita Pradhan, Ramesh S Bhandari

**Affiliations:** 1 Department of Surgical Gastroenterology, Tribhuvan University Teaching Hospital, Maharajgunj Medical Campus, Institute of Medicine, Kathmandu, NPL

**Keywords:** post hepatectomy liver failure, portal vein embolization, liver volume augmentation, extended hepatectomy, bile leak

## Abstract

Background

Extended hepatectomy (≥ 5 liver segments resection) may be required to have a complete surgical resection and provide the best chance of cure of hepatobiliary tumors. It is associated with high morbidity and mortality but with good perioperative care, its outcomes can be improved. This study was conducted to evaluate the early outcomes of extended hepatectomy at a university hospital in Nepal.

Methods

For this study, prospectively collected data from all patients who underwent extended hepatectomy from October 2012 to April 2022 were reviewed and analyzed retrospectively. Demographic data, liver volume augmentation methods used, intraoperative variables, and postoperative complications were analyzed.

Results

Seventeen patients underwent extended hepatectomy from October 2012 to April 2022. Among them 11 (64.7%) were female and the mean age was 53.9 ±16.3 years (18-72 years). Right extended hepatectomy was the most commonly performed procedure (n = 15, 88.2%), and left extended hepatectomy was performed in the remaining (n = 2, 11.8%). Six patients underwent liver volume augmentation procedures (35.3%) with portal vein embolization (PVE) in three, portal vein ligation (PVL) in one, and partial associating liver partition and portal vein ligation for staged hepatectomy (ALPPS) in two patients. Overall complications were 70% with major complications (Clavien Dindo ≥ IIIa) constituting 35.3%. The most common hepatectomy-specific complication was post-hepatectomy liver failure (PHLF) in six cases. The 30-day mortality was 17.6% (three patients).

Conclusion

Extended hepatectomy can be performed with acceptable major complications and mortality rates in selected patients.

## Introduction

The mainstay of treatment of various neoplastic diseases of the liver remains resection [1]. Many hepatobiliary tumors require extended hepatectomy (resection of ≥ 5 liver segments) to achieve a negative resection margin and provide the best chance of cure [2]. Improvements in surgical techniques and intensive care have decreased the morbidity and mortality associated with extended hepatectomy [3]. Preoperative identification of high-risk patients may reduce postoperative morbidity by allowing detailed evaluations and alternative methods to decrease morbidities. Post-hepatectomy liver failure (PHLF) is a frequently encountered complication following extended hepatectomy and can be decreased by proper case selection with an adequate future liver remnant (FLR) [4]. In patients with low FLR, selective use of various liver volume augmentation methods like portal vein embolization (PVE), and associating liver partition and portal vein ligation for staged hepatectomy (ALPPS), can help to increase the FLR and thereby decrease the chances of PHLF and other morbidities associated with low FLR [5].

Studies on outcomes of extended hepatectomy are scarce. The objective of this study was to review our experience with extended hepatectomy and describe the early perioperative outcomes of patients who underwent extended hepatectomy at our center.

The preliminary results of this paper were the subject of an abstract presented as a free paper abstract at the 32nd National Conference of the Indian Association of Surgical Gastroenterology (IASGCON 2022), on 15th October 2022.

## Materials and methods

Prospectively collected data of all patients who underwent extended hepatectomy at the department of surgical gastroenterology from October 2012 to April 2022 were analyzed retrospectively. Ethical approval to conduct the study was obtained from the Institutional Review Committee of the Institute of Medicine with reference number 210(6-11)E2.

Preoperative assessment

The standard preoperative assessment included a detailed history and physical examination, hematological and biochemical investigations including liver function test, hepatitis B/C status, biomarkers (mainly CEA, CA19.9, and AFP), ultrasonography of the abdomen, and contrast-enhanced computer tomography (CECT) of the abdomen with liver volumetric assessment. Magnetic resonance cholangiopancreatography (MRCP) was done in selected patients requiring assessment of biliary tract anatomy like hilar cholangiocarcinoma. Patients presenting with biliary obstruction and cholangitis had biliary drainage using percutaneous transhepatic biliary drainage (PTBD) to decrease the level of bilirubin and resolve cholangitis.

**Figure 1:**
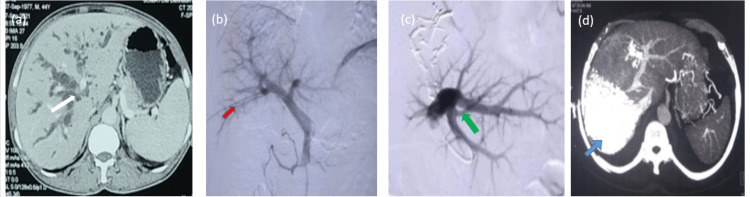
Sequential images of the process of portal vein embolization (PVE). (a) CT scan of the abdomen showing mass at the hepatic hilum (white arrow) causing complete obstruction of the bile duct with dilated intrahepatic biliary radicles. (b) Percutaneous portogram via the right posterior portal vein (red arrow) showing all branches of the portal vein. (c) Selective embolization of the right portal vein and the left medial portal vein with visualization of only the left lateral portal vein (green arrow). (d) Post-procedure CT scan of the abdomen after three weeks of PVE showing hypertrophy of left lobe with atrophic right lobe filled with lipiodol particle (blue arrow) used during PVE.

A standard FLR (sFLR) was calculated in all patients by measuring the ratio of CT-measured FLR with total estimated liver volume (TELV) by using the formula TELV= -794.41 + 1267.28 X BSA) [6]. More than 25% of sFLR was considered safe. Patients with low FLR underwent liver augmentation by either portal vein embolization (PVE) (Fig 1), portal vein ligation (PVL), or partial ALPPS. A repeat CT scan was done in three weeks in cases with PVE and 10 days in cases with partial ALPPS to assess the extent of compensatory liver hypertrophy. A surgical decision was then made based on CT volumetry.

Surgical techniques

Staging laparoscopy was done in selected cases of malignancy (a total of 10 cases, six cases of carcinoma gallbladder, and two cases of hilar cholangiocarcinoma). A modified Makuuchi incision was given in all cases. Complete mobilization of the liver was done and inflow and outflow control were taken. Parenchymal transection was aided by a Hanging maneuver [7]. Transaction techniques (Kelly-clysis, harmonic scalpel, or monopolar cautery) were at the surgeon’s discretion. After completion of the transection, bilioenteric anastomosis was done by Roux en Y hepaticojejunostomy.

**Figure 2:**
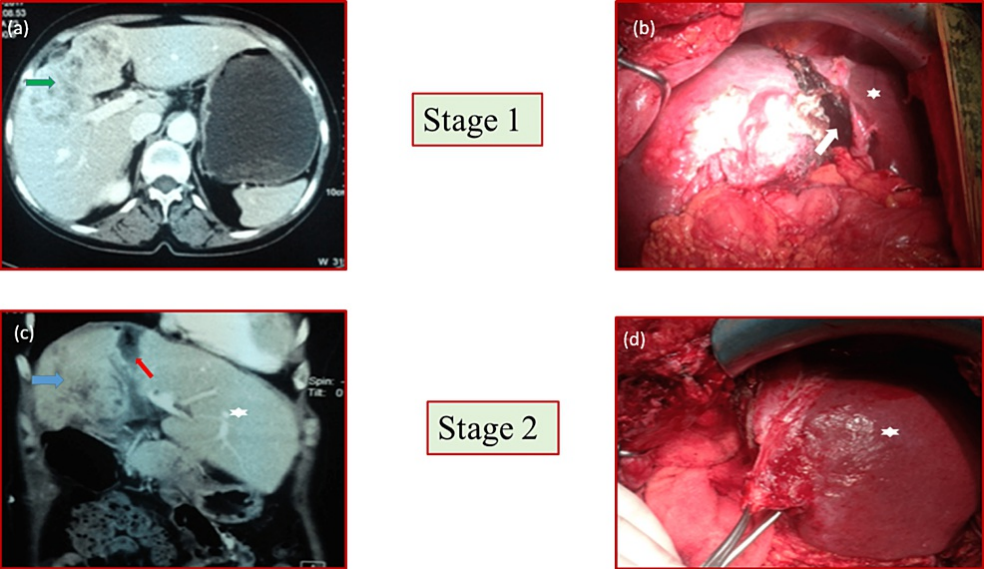
Two stages of partial ALPPS procedure (a) Preoperative CT scan of the abdomen showing a mass in segments IV and V of the liver (green arrow). (b) Partial transection of the liver (white arrow) with ligation of the right portal vein completing stage I. Remnant liver (left lateral sector) marked by the star. (c) CT scan after 10 days of Stage I showing hypertrophy of left lateral sector of the liver (marked by a star) with atrophy of right lobe with mass (blue arrow). The transection line is marked by the red arrow. (d) Completion of stage II with complete transection of liver and Roux en y hepaticojejunostomy. Significant hypertrophy of the remnant can be appreciated compared to (b) marked by the star. ALPPS: associating liver partition and portal vein ligation for staged hepatectomy

For patients undergoing partial ALPPS, first-stage surgery was partial transection of liver parenchyma along with ligation of the right portal vein. Second-stage surgery comprises of completion of the transection with Roux-en-Y hepaticojejunostomy (Figure 2).

Data collection

All variables (demographics, intraoperative, and postoperative) that were recorded in the departmental database were reviewed. Missing data were obtained from hospital record files. The 30-day postoperative outcomes including follow up were reviewed.

Outcomes and definitions

The outcomes measured were overall and hepatectomy-specific complications, a comparison of postoperative outcomes among the liver volume augmentation group versus no augmentation group, and perioperative factors affecting major complications. Hepatectomy-specific complications like PHLF and bile leak were defined as per the International Study Group of Liver Surgery (ISGLS) 2011 [8-9]. Small-for-size syndrome (SFSS) for extended hepatectomy was defined by the University of Heidelberg (2015) [10]. Other complications were defined and graded as per the Clavein Dindo grading system [11]. Perioperative mortality was defined as mortality within 30 days of surgery.

Statistical analysis

We analyzed data using SPSS version 25 (IBM Corp., Armonk, NY). Data were presented in numbers, mean ± SD, and percentages where appropriate. The correlation between variables was analyzed using Fischer’s exact test for categorical variables and sample t-test for continuous variables. A p-value of <0.05 was taken as statistically significant.

## Results

A total of 115 hepatectomies were performed between October 2012 to April 2022, of which 94 (81.7%) had major hepatectomies; 17 (14.7%) extended hepatectomies were performed during this period and all these cases were included in this study. The mean age of the patients was 53.9 ± 16.3 years and a majority of them were females (n = 11, 64.7%). The most common indication was carcinoma gallbladder (n = 6, 35.3%) followed by hilar cholangiocarcinoma (n = 4, 23.5%). The mean preoperative bilirubin was 34.4 ± 22.7 µmol/L. Preoperative biliary drainage in form of PTBD was required in six cases (35.3%). Liver volume augmentation was required in six cases (35.3%) because of low FLR volume; three had PVE, one had PVL, and two had partial ALPPS. The demographic characteristics of the patients are depicted in Table 1.

**Table 1:** Demographics of the patients. * Bilirubin level one day prior to surgery # out of six, three had portal vein embolization (PVE), one had portal vein ligation (PVL), and two had partial associating liver partition and portal vein ligation for staged hepatectomy (ALPPS).

Variables (N=17)	Numbers	Percentage
Age in years (mean ± SD)	53.88 ± 16.32	-
Sex		
Female	11	64.7
Male	6	35.3
Comorbidities	5	29.4
Diagnosis		
Carcinoma gallbladder	6	35.3
Hilar cholangiocarcinoma	4	23.5
Biliary cystadenoma	3	17.6
Intrahepatic cholangiocarcinoma	1	5.9
Hepatocellular carcinoma	1	5.9
Haemangioma	1	5.9
Hepatoblastoma	1	5.9
Bilirubin in umol/L* (mean ± SD)	34.37 ± 22.7	-
Biliary drainage (PTBD)	6	35.3
Volume augmentation techniques	6^#^	35.3

Among the 17 cases, 15 (88.2%) had a right extended hepatectomy, and the other two (11.8%) had a left extended hepatectomy. The mean blood loss was 23.5 ± 219.4 ml (IQR: 500 - 850 ml). The mean operative time was 5.42 ± 1.06 hours (IQR: 5 - 6.45 hours). Major postoperative complications i.e (Clavien Dindo (CD) ≥IIIa, were seen in six (35.3%) of patients (Table 2). Hepatectomy-specific complications like bile leaks were seen in four cases (23.5%), and PHLF was seen in six cases (35.3%) of which three had Grade C PHLF. SFSS was seen in four (23.5%) cases. The mean hospital stay was 17.35 ± 6.2 days. The 30-day mortality rate was 17.6 % (three cases).

**Table 2:** Postoperative complications of extended hepatectomy. PHLF: post-hepatectomy liver failure; SFSS: small-for-size syndrome; SSI: surgical site infection; AKI: acute kidney injury

Variables (N=17)	Number	Percentage
Bile leak	4	23.5
Grade A	2	-
Grade B	1	-
Grade C	1	-
PHLF	6	35.3
Grade A	2	-
Grade B	1	-
Grade C	3	-
SFSS	4	23.5
SSI	3	17.6
Postoperative ileus	1	5.9
Burst abdomen	1	5.9
Pneumonia	1	5.9
AKI	1	5.9
Clavien Dindo Grade		
< IIIa	11	64.7
≥ IIIa	6	35.3

On comparing the outcomes among patients with or without liver volume augmentation (Table 3), bile leaks were significantly high in patients without augmentation (4 vs 0, p = 0.042). PHLF was more common among patients who had volume augmentation (4 vs 2, p = 0.045).

**Table 3:** Comparison between volume augmentation vs no augmentation subgroup. PTBD: percutaneous transhepatic biliary drainage, PHLF: post hepatectomy liver failure, CD: Clavien Dindo

Variables	Volume augmentation (N=6)	No augmentation (N=11)	P-value
PTBD (N)	5	1	0.002
Bilirubin level (umol/L) (mean ± SD)	36.8 ± 24.36	33.02 ± 23.41	0.83
Blood loss (ml) (mean ± SD)	833.3 ± 206.56	663.63 ± 211.05	0.13
Operative duration (hrs) (mean ± SD)	5.32 ± 1.0	5.48 ± 1.19	0.69
Bile leak (N)	0	4	0.042
Grade A	0	2	
Grade B	0	1	
Grade C	0	1	
PHLF (N)	4	2	0.045
Grade A	1	1	
Grade B	1	0	
Grade C	2	1	
CD ≥ IIIa (N)	2	4	0.90
Hospital stay (days) (mean ± SD)	19 ± 6.5	16 ± 6.17	0.44
Mortality (N)	2	1	0.21

Preoperative and Intraoperative factors were assessed for their association with major complications but none of the perioperative variables were significant to cause an effect on the occurrence of major complications (Table 4).

**Table 4:** Factors affecting major complications. PTBD: percutaneous transhepatic biliary drainage

Variables	CD< IIIa (N=11)	CD ≥ IIIa (N=6)	P-value
Age			0.40
<60yrs	6	2	
≥60yrs	5	4	
Gender			0.90
Male	7	4	
Female	4	2	
Pathology			0.91
Benign	4	0	
Malignant	7	6	
Bilirubin (Umol/L)			0.48
<50	9	4	
≥50	2	2	
PTBD			0.35
Yes	3	3	
No	8	3	
Blood Loss (ml)			0.35
<600	3	3	
≥600	8	3	
Volume Augmentation			0.90
Yes	4	2	
No	7	4	

## Discussion

The first anatomical extended right hepatectomy was reported by Lortat Jacob in 1952 [12]. Since then many authors have described the techniques of extended hepatectomies. The extended hepatectomy has evolved over time from a procedure with high mortality of >20% to around 5% in recent studies [13]. The morbidity still remains high at ~50% even in some specialized centers [14]. However, proper case selection and improved perioperative intensive care can decrease the morbidity associated with this procedure.

Makuuchi et al. [15] had recommended a preoperative bilirubin of 52µmol/L (3mg/dl) by routine performing biliary drainage to decrease postoperative morbidity. Hyperbilirubinemia adversely affects the regenerative capacity of the liver. In this study, the mean preoperative bilirubin was 34.37 ± 22.7 µmol/l which is at par with the recommended level. To achieve bilirubin to the desired level, preoperative biliary drainage was done in six cases (35.3%).

Major complications (CD ≥ IIIa) following extended hepatectomy are common. Vauthey et al. [2], had reported a major complication of 28.3% and a mortality of 0.8%. Similar outcomes were also seen by Ubink et al. [16], who reported a major complication of 27% after extended hepatectomy. The higher morbidity and mortality in this study may be attributed to our early experience in this procedure and also to the level of perioperative intensive care in a resource-limited country.

To decrease the risk of PHLF, liver volume augmentation was used in six cases with low FLR. The PHLF rate in a recent study was 20% which is lower than that observed in this study (35.3%) [17]. On subgroup analysis, PHLF was significantly higher in the augmentation group compared to the no augmentation group (4 vs 2, p = 0.045). This high PHLF rate in the augmentation group may be due to the use of partial ALPPS in two cases which is known to have more complications as compared to other methods of volume augmentation [18].

Small-for-size syndrome is considered to be a component of PHLF by many authors and is mainly described in the setting of liver transplantation. But recent studies have indicated it to be an independent entity and is described as an independent complication of extended hepatectomy [10]. The SFSS was seen in 23.5% of cases.

Bile leaks are common after hepatectomies. A study by Ubink et al. [16] on extended hepatectomy showed bile leaks of 3.4%. Similar bile leaks were reported by Vauthey et al [2]. This study has bile leaks of 23.5% (four cases). All bile leaks were seen in cases without volume augmentation. This may be due to the fact that all augmented cases had a preoperative biliary drainage tube in-situ.

There are some limitations in this study. First, it is a retrospective study with a small sample. Due to limited indications of this procedure, it is difficult to conduct a prospective study on extended hepatectomy. Second, oncological outcomes were not studied due to heterogeneous pathology including some benign diseases.

## Conclusions

Extended hepatectomy is a procedure associated with relatively high morbidity and mortality. Proper case selection and good perioperative care can help to decrease the early complication associated with extended hepatectomy. Long-term studies are required in the future to assess the long-term as well as the oncological outcomes of extended hepatectomy.
